# Sex-Specific Effects of Short-Term Oral Administration of Food-Grade Titanium Dioxide Nanoparticles in the Liver and Kidneys of Adult Rats

**DOI:** 10.3390/toxics11090776

**Published:** 2023-09-13

**Authors:** Roberta Tassinari, Alessia Tammaro, Andrea Martinelli, Mauro Valeri, Francesca Maranghi

**Affiliations:** 1 Center for Gender Specific Medicine, Istituto Superiore di Sanità, 00161 Rome, Italy; roberta.tassinari@iss.it (R.T.); alessia.tammaro@iss.it (A.T.); 2 Department of Biomedicine and Prevention, University of Rome “Tor Vergata”, 00173 Rome, Italy; 3 Experimental Animal Welfare Sector, Istituto Superiore di Sanità, 00161 Rome, Italy; andrea.martinelli@iss.it (A.M.); mauro.valeri@iss.it (M.V.)

**Keywords:** osteopontin, vascular endothelial growth factor, interleukin 6, neuropeptide Y, human exposure, hazard identification

## Abstract

Titanium dioxide (TiO_2_) nanomaterial is used in several items (implant materials, pills composition, cosmetics, etc.). Although TiO_2_ is no longer considered safe as a food additive, the general population is exposed daily through different routes, and information is lacking on some aspects of animal and human health. This study evaluated liver and kidney toxicity of food-grade TiO_2_ nanoparticles (NPs) (primary size < 25 nm) in male and female rats that were orally exposed for 5 days to 0, 1, and 2 mg/kg body weight per day (comparable with daily E171 consumption). Selected liver and kidney toxicity endpoints included serum biomarkers, histopathological analysis and expression of osteopontin (SPP1), vascular endothelial growth factor (VEGF), interleukin 6 (IL-6), and neuropeptide Y (NPY). Although TiO_2_ NPs are known to affect the gastric mucosa, short-term exposure induced sex-specific effects: general toxicity parameters were predominantly altered in female rats, whereas the liver appeared to be more affected than the kidneys in male rats, which also showed overexpression of NPY and SPP1. In the kidneys, the TiO_2_ NP effects were quantitatively similar but qualitatively different in the two sexes. In conclusion, careful consideration should be paid to the presence of TiO_2_ NPs in other items that can lead to human exposure.

## 1. Introduction

Titanium dioxide (TiO_2_) nanomaterial (NM) is mainly marketed in two different crystalline forms (anatase or rutile). It is used in several consumer products, for example as implant materials for dental, orthopedic, and osteosynthesis applications [1]. Based on their optical and biological properties, TiO_2_ NMs are also used as bio-compatible materials in high-tech applications, e.g., in UV protection applications [2]. Moreover, TiO_2_ NMs are used in cosmetics, paints, and medicines as an adjuvant in pill composition [3]. The consumption of TiO_2_, as the E171 food additive, has been estimated to be 5–9 mg per person per day, even reaching up to 32.4 mg/kg per day in children [4]. The highest concentrations of TiO_2_ were recorded in chocolate-coated candies, sweets, and chewing gums [5,6]. Recently, the use of TiO_2_ as a food additive was considered no longer safe by the European Food Safety Authority (EFSA) [7], and the European Commission announced the decision to ban its use starting in August 2022 [8]. Thus, considering the wide range of applications, the general population can be exposed to TiO_2_ NMs daily through different routes (inhalation, ingestion, dermal penetration, and injection), but there is still a lack of information on some aspects of animal and human health, and on the impact on the environment. Several data are available on TiO_2_ NM exposure through inhalation or the dermal route: the inhalation of fine TiO_2_ particles, and especially nanoparticles (NPs), are regarded as critical to health, as studies with animals have shown that they can penetrate deep into the lungs, and might cause chronic inflammations leading to lung tumors [3]. Moreover, it has been suggested that food-grade TiO_2_ may initiate and promote the development of colon preneoplastic lesions in rats [9]. Indeed, the oral route of exposure remains poorly investigated compared to the dermal route or inhalation. Previous data from the same study showed sex-specific effects, with increased length of intestinal villi and serum testosterone levels in male rats, and the mass spectrometry analysis confirmed NP deposition in intestinal tissues [10]. Moreover, histological alterations were observed in the thyroid, adrenal medulla cortex, and ovarian granulosa of female rats, with increased total TiO_2_ tissue levels in the spleen and ovaries, whereas reduced T3 was observed in male rats only [11]. Additionally, one study pointed out that, in rodents administered by oral route, tissue deposition occurred predominantly in the liver and kidneys [12]. However, few in vivo studies have focused on liver and kidney toxicity, or on the potential sex-specificity of the effects [13,14].

The aim of this study was to determine the liver and kidney toxicity of food-grade TiO_2_ NPs in male and female rats orally exposed to dose levels comparable with the daily consumption of E171. Selected liver and kidney toxicity endpoints included serum biomarkers and histopathological analyses, as well as the expression of a panel of specific genes evaluated in both tissues.

Osteopontin (SPP1), which is overexpressed in various malignancies promoting proliferation, migration, and invasion, is normally expressed in the bile duct epithelium, and represents a suitable, early diagnostic marker for hepatocarcinoma [15]. In renal tissues, SPP1 has been correlated with proteinuria, reduced creatinine clearance, and kidney fibrosis in animal models of kidney diseases [16]. In the liver, vascular endothelial growth factor (VEGF) is the main regulator of angiogenesis, and its expression was correlated with bile duct growth [15]. In the kidney, VEGF, produced by glomerular epithelial cells, regulates ultrafiltration; nevertheless, its role in the tubule is poorly understood [17]. The inflammatory cytokine interleukin-6 (IL-6) exerts multiple functions in the body, and contributes to acute and chronic kidney injury and fibrosis [18]. In the liver, IL-6 is crucial for hepatocyte homeostasis, and is a potent hepatocyte mitogen. In addition, IL-6 is implicated in metabolic function [19].

Neuropeptide Y (NPY) is expressed in the neurons of the brain and in abdominal organs including the liver, and its biological function is the regulation of appetite and body weight homeostasis. In this respect, NPY is involved in metabolic functions [20]. Moreover, NPY is a co-transmitter in the vasculatures of many organs, and it shows potent vasoconstriction. Indeed, NPY may exert renal vasoconstriction and tubular actions in a species-specific way, with the capacity to alter renal function and renin secretion [20,21].

## 2. Materials and Methods

### 2.1. Sample Characterization

TiO_2_ NPs (CAS number 1317-70-0, anatase, nanopowder, primary size < 25 nm, BET surface area 45–55 m^2^/g, 99.7% trace metals basis) were purchased from Sigma-Aldrich Company Ltd. (Gillingham, Dorset, UK). Sample and dispersion characterization for in vivo study was performed according to the procedure described in [10,11]. Briefly, two milligrams of TiO_2_ NP were weighed and suspended in ultrapure water. The stock suspensions were sonicated with a probe sonicator (Vibracell, Sonics & Materials Inc., Newtown, CT, USA) at 750 W, 20 KHz, 20% amplitude, and 6.5 mm probe diameter, in order to reduce agglomeration. Primary size, shape, size distribution, and agglomeration status of TiO_2_ NP suspensions were determined using TEM (EM 208, FEI Company, Amsterdam, The Netherland), scanning electron microscope (SEM) (FE-SEM Quanta Inspect, FEI Company, Amsterdam, The Netherlands) equipped with Soft Imaging System and by dynamic light scattering (DLS; Zetasizer Nano, Malvern Instruments, London, UK) [10,11].

### 2.2. Animals and Treatment

The study was carried out in compliance with the ARRIVE guidelines and the 3R rules. Every effort was made to minimize the group sample size and the suffering of the animals used in the experiment. 30 young sexually mature Sprague Dawley male and female rats, approximately 60 days old, were purchased from Envigo (Milano, Italy). Upon arrival, all the animals were housed in the same room in transparent Plexiglass cages, and they were kept under standard laboratory conditions (room temperature, i.e., 22 ± 0.5 °C, 50–60% relative humidity, 12 h dark–light cycle with 12–14 air changes per hour) at the animal facility of Istituto Superiore di Sanità [11]. Tap water and pellet food were available ad libitum. After five days of acclimatization, the rats were divided into three treatment groups (5 rats/sex/group):

(1) Control, vehicle only (distilled water);
(2) 1 mg/kg bw per day of TiO_2_ NPs;
(3) 2 mg/kg bw per day of TiO_2_ NPs.

Rats were treated per os by gavage for five consecutive days, from treatment day (TD) 1 to 5. The dose levels were selected in the range of daily consumption of E171 as NM, taking into account that the maximum volume administered by gavage to each rat cannot exceed 2 mL/100 g bw in aqueous solution [22]. Body weight and food consumption were recorded daily. TiO_2_ NPs were suspended in distilled water by sonication for 15 min, and the fresh dispersions were prepared on a daily basis. Twenty-four hours after the last treatment (TD 6), male and female rats were anaesthetized with a gaseous solution of isoflurane and blood samples were collected by intracardiac puncture for the determination of the liver and kidney serum biomarkers. Subsequently, the animals were sacrificed by CO_2_ inhalation. The liver and kidneys were excised and weighed for histopathological examination and gene expression analysis.

### 2.3. Serum Biomarkers

After collection, blood samples were left to coagulate at room temperature for 1 h, centrifuged for 15 min at 2000 rpm twice in a cooled bench-top centrifuge (Microlite Microfuge, Thermo Electron Corporation, Waltham, MA, USA) and stored at −80 °C until use. Alanine aminotransferase (ALT) and aspartate aminotransaminase (AST) as markers of liver toxicity, creatinine (CREA), and blood urea nitrogen (BUN) as markers of kidney toxicity were measured in serum of treated and control rats of both sexes by the automatic analyzer Keylab Analyser (BPC Biosed S.R.L., Milano, Italy) [23].

### 2.4. Histological and Histomorphometrical Analysis

Immediately after the sacrifice, to avoid any possible post-mortem artefacts, the liver and kidneys were fixed in 10% buffered formalin and stored in 80% ethyl alcohol. They were dehydrated in a graded series of alcohol baths and embedded in paraffin by tissue processor (Shandon Excelsior ES, Thermo Scientific, Waltham, MA, USA). The histological sections of 5 μm thick were prepared using the Microm. HM 325 (Thermo Scientific, Waltham, MA, USA) and stained with hematoxylin/eosin for the examination under a light microscopy (Nikon Microphot FX, Melville, NY, USA) at various magnifications to evaluate the histopathological alterations.

The quantitative histomorphometrical analyses were performed on kidneys. The transverse section was examined by means of an image analysis system (Nis-Elements BR) applied to an optical microscope (Nikon Microphot FX, Melville, NY, USA). The diameter and area were measured for 100 glomeruli/sample using a 10× lens, and the glomerular volume was calculated [24].

### 2.5. Gene Expression Analysis

Total RNA was extracted from paraffin-embedded samples of liver and kidney (5 samples/treatment group) using the RNeasy FFPE Kit (Cat. No./ID: 73,504; QIAGEN, Düsseldorf, Germany) according to the manufacturer’s instructions. Briefly, paraffin was removed from 10 μm thick sections by Bio Clear (Bio-Optica, Milano, Italy), then proteinase K tissue digestion was performed (10 μm; 56 °C for 15 min). To reverse formalin crosslinking, samples were incubated at 80 °C for 15 min. Genomic DNA was removed by DNase digestion, RNA was quantified by Nabi Nano Spectrophotometer (MicroDigital Co., Ltd., Seoul, Republic of Korea) and ran on 1% agarose to evaluate its integrity. Despite poor yield, all the samples met the quality criteria (A260/A280 ≥ 1.8) to proceed with real-time PCR analysis. cDNA synthesis and qRT-PCR were performed [25]. The list of primers is provided in Supplementary Materials (Table S1). Glyceraldehyde-3-phosphate dehydrogenase (GAPDH) was used as a control gene for RNA normalization. The relative amount of each substrate was calculated by the 2−∆∆Ct method.

### 2.6. Data Analysis

Data management was performed using Microsoft Excel 2013 for Windows 10. All data were entered by a single blinded operator, who also performed all the statistical analyses, and they were analyzed using the software JMP 10 (SAS Institute Inc., Cary, NC, USA). GraphPad Prism 6.0 software was used to perform all graphics. Data were presented as mean ± standard deviation (SD). A non-parametric Kruskal–Wallis analysis was performed to analyze data, followed by post hoc pairwise comparisons (Mann–Whitney test).

Quantal data were analyzed by one-tailed Fisher’s Mid-P test to assess significant differences with respect to control group. The Cochran–Armitage Trend Test was performed to evaluate the dose–response trend. Differences among groups were considered significant if the *p*-value was <0.05.

## 3. Results

### 3.1. Characterization of TiO_2_ NP Suspensions

**Figure 1:**
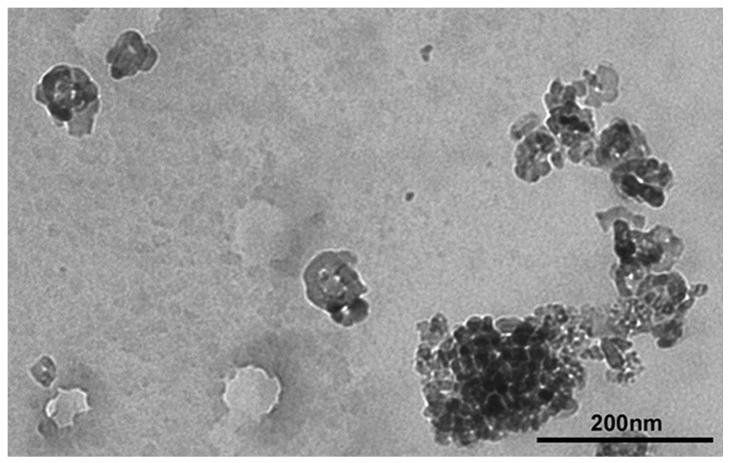
TEM image of TiO_2_ NPs [10,11].

The main results are summarized in Table 1. TEM analysis indicated two morphologies for water suspension of TiO_2_ NPs: spherical shape (primary size from 20 to 60 nm) and irregular shape (length of about 60 nm and width of about 40 nm) with different size agglomerates, up to 200 nm of length (Figure 1) [10]. SEM analysis identified an average diameter between 70 nm and 1.2 μm with a peak between 60 nm and 90 nm and, considering all TiO_2_ NPs analyzed, 13% showed dimensions below 100 nm [10,11].

### 3.2. General Toxicity

No deaths or clinical effects have been recorded in either male or female rats.

**Figure 2:**
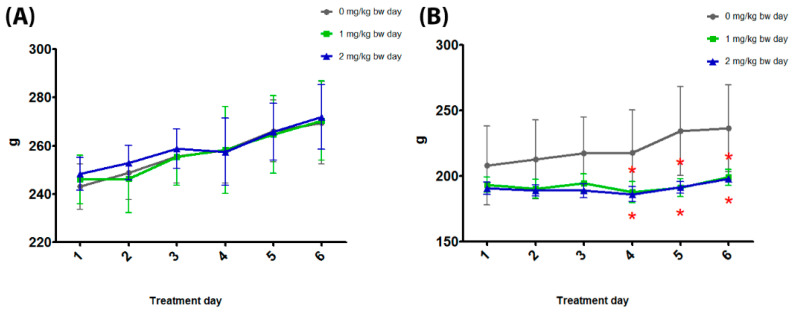
Body weight of male (**A**) and female (**B**) rats orally treated for 5 days with 0, 1, and 2 mg/kg bw per day of TiO_2_ NP. * *p* < 0.05.

The body weight was significantly reduced at 1 and 2 mg/kg bw in female rats at treatment days 4, 5, and 6. The body weight of male rats was unaffected (Figure 2 and Table 2).

During the treatment, the mean of food consumption was significantly reduced at 1 and 2 mg/kg bw in female and at 2 mg/kg bw in male rats (Table 2). The relative kidney weight was significantly increased at 2 mg/kg bw in female and at 1 and 2 mg/kg bw in male rats (Table 2). The absolute kidney weight, and absolute and relative liver weights, were unaffected in both sexes (Table 2).

### 3.3. Serum Biomarkers

#### 3.3.1. Liver

Serum levels of AST and ALT were unaffected in both sexes (Figure 2).

#### 3.3.2. Kidneys

**Figure 3:**
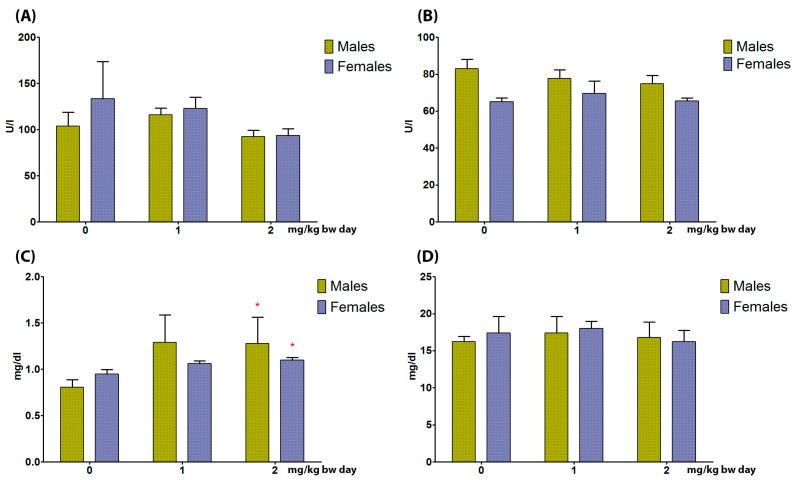
Aspartate aminotransaminase (**A**), alanine aminotransferase (**B**), creatinine (**C**), and blood urea nitrogen (**D**) serum levels in male and female rats orally treated for 5 days with 0, 1, and 2 mg/kg bw per day of TiO_2_ NP Kruskal–Wallis * *p* < 0.05. (*n* = 5).

CREA serum levels were significantly increased at 2 mg/kg bw in male and female rats. Serum levels of BUN were unaffected (Figure 3).

### 3.4. Histopathological Analysis

#### 3.4.1. Liver

**Figure 4:**
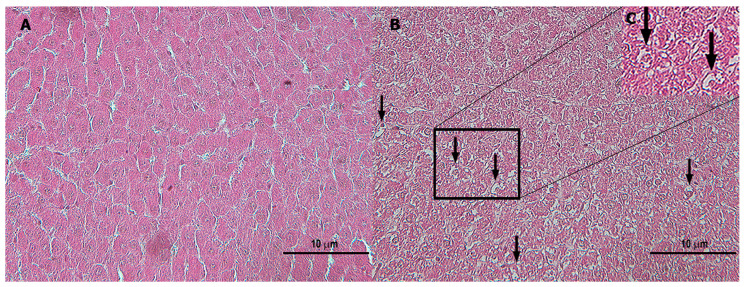
Light microscope image of liver of control and treated rats. (**A**) Control group showing normal hepatocytes; (**B**,**C**) hepatocyte vacuolization (black arrow) 2 mg/kg bw day of TiO_2_ NP male rats. Magnification: 20×. Scale bar: 10 μm. H and E staining.

A significant increase in hepatocyte vacuolization/steatosis at 2 mg/kg bw (Figure 4) and a dose-dependent increase in intralobular lymphoid infiltration and congestion in the central vein with enlargement of the sinusoids significantly at 1 and 2 mg/kg bw were present in male rats in comparison to control group (Table 3).

In female rats, hepatocyte vacuolization/steatosis and intralobular lymphoid infiltration were significantly increased at 1 and 2 mg/kg bw compared to control group. In addition, a dose-dependent significant increase in congestion in the central vein with enlargement of the sinusoids and focal intralobular necrosis in the middle zone of the liver lobule at 2 mg/kg bw in comparison to control group was present (Table 3).

#### 3.4.2. Kidneys

**Figure 5:**
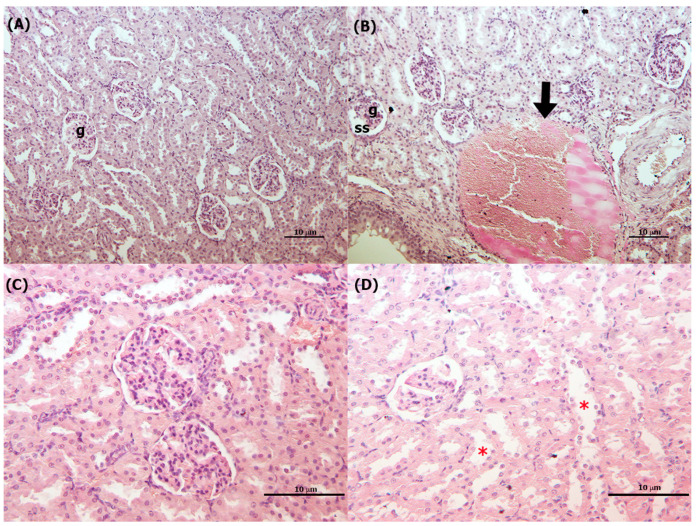
Light microscope image of kidney tissues of control and treated rats. (**A**) Control group shows the intact architecture of the renal cortex, with normal size glomeruli and small subcapsular spaces; (**B**) smaller glomeruli, dilated subcapsular space and vascular congestion and dilatation (black arrow), male rat, 2 mg/kg bw day of TiO_2_ NP. (**C**) Control group shows the intact architecture of the renal cortex with normal tubules. (**D**) Dilated tubules (red stars), female rat, 2 mg/kg bw day of TiO_2_ NP (g: glomeruli; ss: subcapsular spaces). Magnification: 10× (**A**,**B**), 20× (**C**,**D**), scale bar: 10 μm. H and E staining.

A significant increase in vascular congestion and dilatation at 1 and 2 mg/kg bw in comparison to control group was present in male rats (Table 3; Figure 5). In female rats, a significant increase in renal tubule dilatation with cellular desquamation was observed at 1 and 2 mg/kg bw in comparison to control group (Table 3; Figure 5), and a significant increase in vascular congestion and dilatation at 2 mg/kg bw was observed, both parameters are dose-dependent (Table 3). A significant reduction in glomerular area, diameter, and volume at 2 mg/kg bw was recorded in male rats (Table 3)

### 3.5. Gene Expression Analysis

#### 3.5.1. Liver

**Figure 6:**
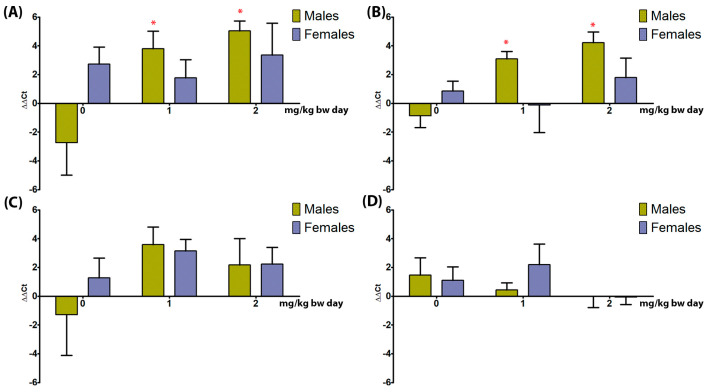
Gene expression analysis of liver NPY (**A**), SPP1 (**B**), IL6 (**C**), and VEGFA (**D**) by real-time PCR in male and female rats orally treated for 5 days with 0, 1, and 2 mg/kg bw per day of TiO_2_ NP. Data are presented as mean ∆∆Ct values ± standard deviation, with control samples as calibrators and GAPDH as the reference gene. Statistical significance: * *p* < 0.05 Mann–Whitney test. *N* = 5.

In male rats, NPY and SPP1 were significantly upregulated in all treatment groups, whereas in female rats, they were unaffected (Figure 6A,B). IL6 and VEGFA were unaffected in both sexes (Figure 6C,D).

#### 3.5.2. Kidneys

NPY, SPP1, IL6, and VEGFA were unaffected in both sexes.

## 4. Discussion

TiO_2_ NPs as the E171 food additive are no longer considered safe for human consumption; indeed, TiO_2_ NPs are still present as main component in several product of daily use, thus justifying the in-depth analysis of mechanisms and pathways in target organs, at dose levels and oral administration both relevant for human exposure. Previous data showed that endocrine-active tissues are target of the same TiO_2_ NPs [11], as well as that they are involved in proliferative processes at the gut level, in both in vitro and in vivo studies [10]. The present paper is focused on the liver and kidneys as primary target organs of detoxification and excretion, which are the most prone to potential NP effects. Indeed, several studies identify the liver as a target of TiO_2_ NP toxicity due to its metabolic competence, showing that TiO_2_ NPs accumulate in the liver with slow excretion, above all NPs of small sizes for which clearance is very difficult [14,26,27]. Interestingly, the administration of food-grade TiO_2_ NP at dose levels close to the estimated human exposure through food induced liver effects more marked in male rats, including the expression of alternative pathways of TiO_2_ NP toxicity focused on proliferation and vascular growth. Indeed, to our best knowledge, this is the first study that identifies NPY and SPP1 sex-specific alterations induced by NPs. It should be noted that the study evaluated the expression of genes at the level of mRNA, and that the data were not confirmed by the protein expression; this will represent the aim of further in deep analyses. Concerning general toxicity data, TiO_2_ NP suspension, as already observed, induced direct toxic effects on gastric mucosa in female rats and, to a lesser extent, in males [28]. Histopathological analysis showed steatosis was accompanied, in males only, by up-regulation of NPY expression at both dose levels. NPY is known to be involved in adipose tissue inflammation and liver steatosis [29]. Indeed, steatosis was more marked in female rats, but NPY expression was unaltered. It can be hypothesized that a different, more marked sensitivity of the female liver to TiO_2_ NP after oral ingestion is independent from NPY action, as described by Chen Z et al. (2019) [26]. Interestingly, upregulation of SPP1 was also present at both dose levels in male rats only. SPP1, also known as phosphoprotein 1, is a glycoprotein involved in the immune and inflammatory responses, playing an active role in the development of diabetes, fatty liver disease, and cancer [30]. In female rats, SPP1 expression is regulated by estrogens, their lower levels having protective effect towards liver injuries, whereas physiologically higher levels have no effects [31]. This can explain the different pattern of effects recorded in male and female rats. The livers of both sexes showed other changes in hepatocytes that might be due to direct TiO₂ NP injury, leading to metabolic and structural disturbances [32]. Although congestion in the central vein and enlargement of the sinusoids were recorded, VEGFA gene expression did not show any alteration. The administration of 100 mg/kg bw of TiO_2_ NP (21 nm) by gavage for 30 days to male rats induced the same, although more marked, alterations, probably due to the higher dose level and the longer exposure time [33]. Considering the intralobular lymphocyte accumulation, this can be seen as a normal condition, part of the process of hepatic immune surveillance [34]. In the present study, the accumulation was present only in treated rats, identifying a response to injury induced by TiO_2_ NP; again, this effect appeared to be more evident in male rats. As concerns IL-6 gene expression, it was not modulated by the treatment in both sexes, indicating that that IL is not involved in the liver toxicity mediated by TiO_2_ NPs. Hepatic vacuolization and inflammatory cell infiltration were present in male rats orally treated with TiO_2_ NPs (size 75 ± 15 nm) at dose levels from 10 to 200 mg/kg bw per day for 30 days [35]. It is interesting to note that several data on TiO_2_ NPs are obtained in male rats, and this makes difficult to perform a reliable comparison with female data, as in the present study, and to highlight sex-specific effects. The lack of alterations reported in the functional biomarkers, as well as the lack of Ti concentration at tissue level [11], may be related to the short-term exposure and low dose levels. Indeed, oral administration of TiO_2_ NPs at 0, 2, 10, and 50 mg/kg bw for 90 days in male rats likewise did not induce effects on ALT and AST at 2 mg/kg bw in both sexes [26], whereas male rats orally exposed to 300 mg/kg TiO2 NPs (20 nm) for three weeks showed significantly increased serum levels of both ALT and AST [27]. The same was seen in female mice treated with anatase TiO_2_ NPs (62.5, 125, 250 mg/kg bw—5 nm) by intragastric administration for 30 days [36].

The kidneys are crucial to regulate body homeostasis and the excretion of several molecules. After systemic exposure, a higher accumulation of TiO_2_ NPs in the kidneys, as compared with the spleen and the liver, has been reported [37] and, although TiO_2_ NPs could be eliminated both in urine and feces, the urinary excretion rate is higher [38]. It was already reported that TiO_2_ NPs induce morphological and physiological alterations in the kidneys, but the different experimental conditions (e.g., species, routes, time and dose levels of administration, and type of TiO_2_ NPs) limit drawing conclusions on the effects of TiO_2_ NP exposure in kidneys [39]. In the present study, relative weight and CREA serum levels, the main biomarker of kidney injury, were increased in both sexes at 2 mg/kg. It is interesting to note that VEGF expression, which regulates ultrafiltration, the main renal activity in kidneys, is not altered by the treatment, indicating that the toxic effect is mediated by different mechanisms not involving VEGF. TiO_2_ NP (20 nm) administered to male rats by gavage for 3 weeks at 300 mg/kg also induced a significant increase in renal CREA [40]. No comparative data are present for female rats; on the contrary, in female mice treated with 5, 10, 50, 100, and 150 mg/kg bw TiO_2_ NPs (anatase) for 14 days by IP injection, no changes in CREA levels were recorded [41]. The sex-specific renal injury is confirmed by the histopathological data; in fact, male rats showed subtle alterations in glomerular dimensions (2 mg/kg bw) and vascular congestion/dilatation, while in female rats, tubular and vascular alterations predominate. Glomerular wrinkling was recorded in male rats orally treated with 300 mg/kg bw of TiO_2_ NPs (20 nm) [40], and vascular dilatation was also evident in male rats treated once with 50 mg/kg bw by SC injection of TiO_2_ NPs (21 nm) [42], and in male rats orally treated for 30 days with 100 mg/kg bw TiO_2_ NPs (21nm) [33]. On the other hand, male rats treated by IP injection with four different doses of TiO_2_ NPs (52 ± 15 nm) 0.5, 1, 4, and 16 g/kg bw, and euthanized 4 days, 1 month, and 2 months after the end of the treatment, did not show any remarkable kidney effects, maybe due to the different NP dimensions and routes of exposure [12].

The importance of NPY in the regulation and protection of kidney function has been debated for a long time [21,43], but no definitive answer has been found yet; the present study did not substantially contribute, at least for TiO_2_ NP involvement, since NPY was nor altered by the treatment in both sexes. Moreover, as for the liver, IL-6 gene expression was not modulated by the treatment in both sexes, indicating that this IL is not involved in the kidney toxicity mediated by TiO_2_ NPs.

## 5. Conclusions

It is known that the liver and kidneys are particularly susceptible to many toxic compounds, including metals and their NPs, both for the high blood supply and their ability to concentrate toxins; moreover, TiO_2_ NPs, in different ways according to their physico-chemical characteristics, remain in contact with hepatic and renal structures longer, and this represents and additional reason for their toxicity. The short-term administration of food-grade TiO_2_ NPs at dose levels comparable to human exposure induce sex-specific effects on liver and kidneys; the liver appeared to be more affected, above all, in male rats, whereas in the kidneys, the TiO_2_ NP effects are quantitatively similar, but qualitatively different, in male and female rats.

In conclusion, the present study contributed to providing data for TiO_2_ NP hazard identification; in fact, although E171 food additive-containing NPs are no longer considered safe for human consumption and their use is consequently banned, careful considerations should be done for the presence of TiO_2_ NPs in other items that can lead to human exposure.

## Figures and Tables

**Table 1:** Characterization of TiO_2_ NP suspensions. TEM: transmission electron microscopy; SEM: scanning electron microscope; DLS: dynamic light scattering.

Parameters/ Characterization Method	Results
Shape/TEM	Spherical, primary size 60–40 nm and irregular length about 60 nm, with about 40 nm width
Size distribution/SEM	Average diameter 70 nm–1.2 μm with a pick from 60 to 90 nm
Z-Average/DLS	604 ± 24 nm
Polydispersity Index/DLS	0.211 ± 0.035

**Table 2:** General toxicity data of male and female rats orally treated for 5 days with 0, 1, and 2 mg/kg bw per day of TiO_2_ NP. Kruskal–Wallis * *p* < 0.05. M: mean. SD: standard deviation. *n* = 5.

		Male			Female	
	0 mg/kg	1 mg/kg	2 mg/kg	0 mg/kg	1 mg/kg	2 mg/kg
Body weight TD6 (g; M ± SD)	275.90 ± 13.57	274.36 ± 14.57	277.18 ± 8.96	236.46 ± 33.63	198.96 ± 6.67 *	197.78 ± 2.72 *
Food consumption (g/day; M ± SD)	23.66 ± 1.85	26.37 ± 1.71	16.98 ± 1.00 *	18.98 ± 1.50	16.54 ± 1.08 *	16.46 ± 0.97 *
Liver absolute weight (g; M ± SD)	13.31 ± 1.14	12.79 ± 0.61	13.07 ± 0.58	8.53 ± 0.50	8.76 ± 0.26	8.78 ± 0.48
Liver relative weight (×100; M ± SD)	4.82 ± 0.23	4.82 ± 0.16	4.91 ± 0.09	4.16 ± 0.29	4.48 ± 0.20	4.47 ± 0.21
Kidneys absolute weight (g; M ± SD)	1.88 ± 0.07	1.94 ± 0.12	1.89 ± 0.03	1.40 ± 0.05	1.34 ± 0.09	1.40 ± 0.08
Kidneys relative weight (×100; M ± SD)	0.69 ± 0.01	0.74 ± 0.02 *	0.75 ± 0.04 *	0.66 ± 0.03	0.71 ± 0.06	0.78 ± 0.04 *

**Table 3:** Histopathological endpoints analyzed in liver and kidney of male and female rats orally treated for 5 days with 0, 1, and 2 mg/kg bw per day of TiO_2_ NP. Fisher’s Mid-P Test * *p* < 0.05; ** *p* < 0.01; Cochran–Armitage Trend Test § *p* < 0.05; §§ *p* < 0.01. *n* = 5.

		Male			Female	
	0 mg/kg	1 mg/kg	2 mg/kg	0 mg/kg	1 mg/kg	2 mg/kg
**Liver**						
Hepatocyte vacuolization/steatosis	0/5	2/5	3/5 *	0/5	4/5 *	3/5 *
Intralobular lymphoid infiltration	0/5 §§	4/5 *	5/5 **	0/5	5/5 **	2/5
Congestion in central vein with enlargement of sinusoids	0/5 §§	4/5 *	5/5 **	0/5 §§	2/5	5/5 **
Focal intralobular necrosis in the middle zone of liver lobule	0/5	0/5	0/5	0/5 §	1/5	4/5 *
**Kidney**						
Tubule dilatation	1/5	0/5	1/5	0/5 §§	3/5 *	5/5 *
Vascular congestion and dilatation	1/5	5/5 *	4/5 (*p* = 0.0537)	0/5 §	2/5	4/5 *
Glomerular area (μm^2^)	7773.7 ± 753.2	7235.6 ± 111.1	6584.2 ± 395.6 *	6236.4 ± 382.4	5849.3 ± 598.3	6086.2 ± 353.7
Glomerular diameter (μm)	93.6 ± 4.6	91.4 ± 3.2	84.1 ± 3.1*	87.6 ± 5.3	79.3 ± 6.0	82.3 ± 2.5
Glomerular volume (μm^3^)	965,817.4 ± 40,089.0	854,634.5 ± 19,851.1	751,084.6 ± 66,154.5 *	692,813.0 ± 63,609.5	630,580.6 ± 97,728.9	667,862.4 ± 58,424.3

## Data Availability

Not applicable.

## Supplementary Materials

The following supporting information can be downloaded at: https://www.mdpi.com/article/10.3390/toxics11090776/s1. Table S1. The primer sequences for analyzed genes. NPY: Neuropeptide Y; SPP1: Osteopontin; IL6: interleukin-6; VEGFA: vascular endothelial growth factor. Figure S1. Gene expression analysis of kidney NPY (A), SPP1 (B), and IL6 (C) and VEGFA (D) by real-time PCR of male and female rats orally treated for 5 days with 0, 1, and 2 mg/kg bw per day of TiO2 NPs. Data are presented as mean ∆∆Ct values ± standard deviation, with control samples as calibrators and GAPDH as the reference gene. Statistical analysis: Mann–Whitney test. n = 5.
