# Supplementary material for: Sex-Specific Effects of Short-Term Oral Administration of Food-Grade Titanium Dioxide Nanoparticles in the Liver and Kidneys of Adult Rats
Source: Toxics. 2023 Sep 13;11(9):776. doi: 10.3390/toxics11090776 (PMC10536411; doi:10.3390/toxics11090776)
Supplement: Supplementary file 1 [file toxics-11-00776-s001.zip › toxics-2552096-supplementary.pdf]

Supplementary Material

Table S1 The primer sequences for analysed genes. NPY: Neuropeptide Y; SPP1: Osteopontin; IL6: interleukin-6; VEGFA: Vascular Endothelial Growth Factor

Gene	Primer	Sequence (5'-3')
NPY	forward	CCCGCCATGATGCTAGGTAA
	reverse	AGTGTCTCAGGGTGGATCT
SPP1	forward	CCAGCCAAGGACCAACTACA
	reverse	AGTGTTTGCTGTAATGCGCC
IL6	forward	CTCTCCGCAAGAGACTTCCAG
	reverse	TTCTGACAGTGCATCATCGCT
VEGFA	forward	CGGTTCCAGAAGGGAGAGGA
	reverse	ACTTCACCACTTCATGGGCT

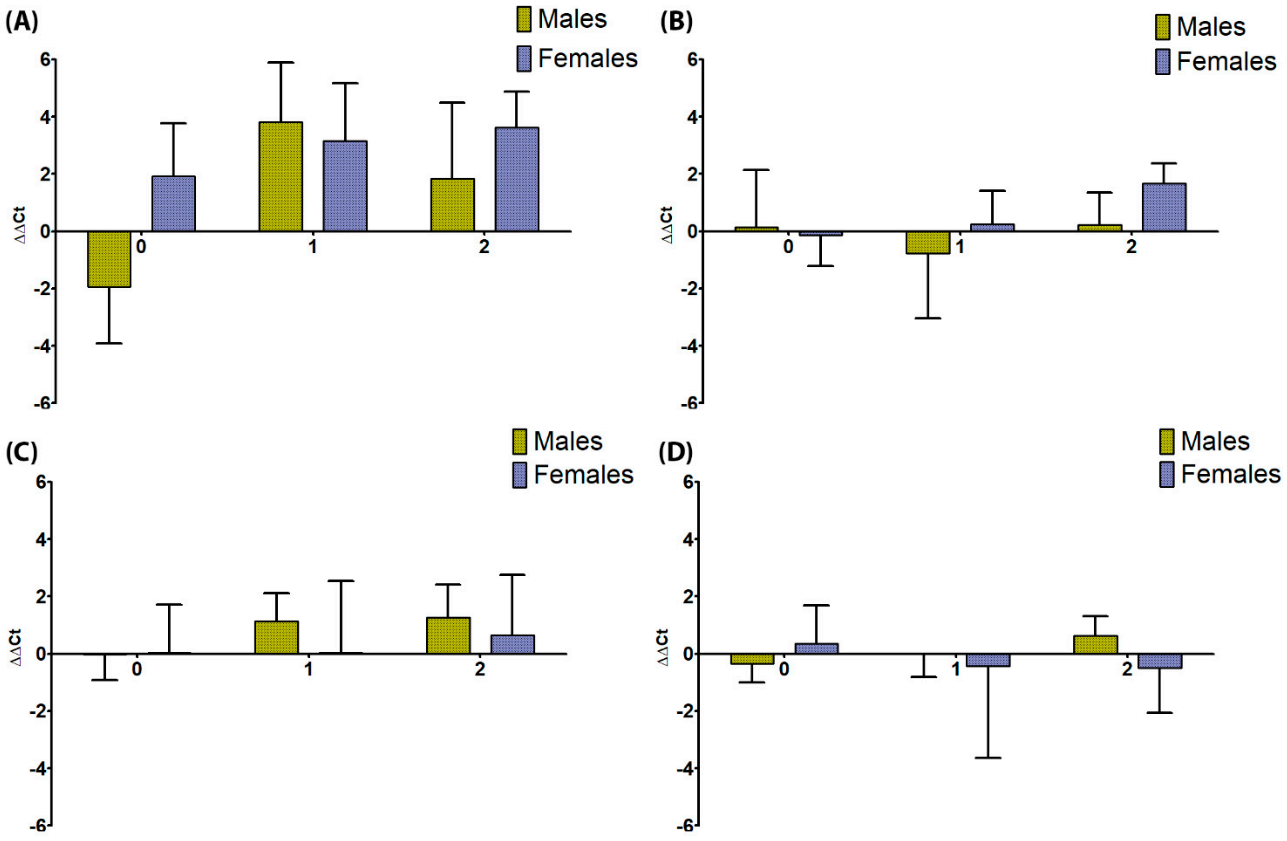

Figure S1. Gene expression analysis of kidney NPY (A), SPP1 (B), and IL6 (C) and VEGFA (D) by real-time PCR of male and female rats orally treated for 5 days with 0, 1 and 2 mg/kg bw per day of TiO_2 NP. Data are presented as mean ΔΔCt values ± standard deviation, with control samples as calibrators and GAPDH as the reference gene. Statistical analysis Mann–Whitney test. n=5.
